# Mobile Smartphone-Based Digital Pupillometry Curves in the Diagnosis of Traumatic Brain Injury

**DOI:** 10.3389/fnins.2022.893711

**Published:** 2022-07-01

**Authors:** Lynn B. McGrath, Jessica Eaton, Isaac Joshua Abecassis, Anthony Maxin, Cory Kelly, Randall M. Chesnut, Michael R. Levitt

**Affiliations:** ^1^Department of Neurological Surgery, University of Washington, Seattle, WA, United States; ^2^Department of Orthopedics and Sports Medicine, University of Washington, Seattle, WA, United States; ^3^Department of Global Health, University of Washington, Seattle, WA, United States; ^4^Department of Radiology, University of Washington, Seattle, WA, United States; ^5^Department of Mechanical Engineering, University of Washington, Seattle, WA, United States; ^6^Stroke and Applied Neuroscience Center, University of Washington, Seattle, WA, United States

**Keywords:** traumatic brain injury, pupillary light reflex (PLR), machine learning, mobile technology, pupillometry

## Abstract

**Objective:**

The pupillary light reflex (PLR) and the pupillary diameter over time (the PLR curve) is an important biomarker of neurological disease, especially in the diagnosis of traumatic brain injury (TBI). We investigated whether PLR curves generated by a novel smartphone pupillometer application could be easily and accurately interpreted to aid in the diagnosis of TBI.

**Methods:**

A total of 120 PLR curves from 42 healthy subjects and six patients with TBI were generated by PupilScreen. Eleven clinician raters, including one group of physicians and one group of neurocritical care nurses, classified 48 randomly selected normal and abnormal PLR curves without prior training or instruction. Rater accuracy, sensitivity, specificity, and interrater reliability were calculated.

**Results:**

Clinician raters demonstrated 93% accuracy, 94% sensitivity, 92% specificity, 92% positive predictive value, and 93% negative predictive value in identifying normal and abnormal PLR curves. There was high within-group reliability (*k* = 0.85) and high interrater reliability (*K* = 0.75).

**Conclusion:**

The PupilScreen smartphone application-based pupillometer produced PLR curves for clinical provider interpretation that led to accurate classification of normal and abnormal PLR data. Interrater reliability was greater than previous studies of manual pupillometry. This technology may be a good alternative to the use of subjective manual penlight pupillometry or digital pupillometry.

## Introduction

Traumatic brain injury (TBI) is a major contributor to global morbidity and mortality. In 2016, there were an estimated 27.1 million new cases of TBI (a rate of approximately 369 per 100,000 people globally), causing an associated 8.1 million years lived with disability (YLD) (Alali et al., 2018). The National Study on the Costs and Outcomes of Trauma demonstrated that up to 60% of severe TBI patients are under-triaged and admitted to non-trauma hospitals (Capone-Neto and Rizoli, 2009; Carrick et al., 2021). This pattern of under-triage results in an excess mortality of 25% for TBI patients in the United States (Chesnut et al., 1993; Capone-Neto and Rizoli, 2009; Chen et al., 2011; Cheng et al., 2017; Carrick et al., 2021). Excess mortality is potentially higher for low and middle-income countries where 85% of the world’s population resides, and where mortality is doubled following severe TBI (Couret et al., 2016; Alali et al., 2018; Dagain et al., 2018; Dewan et al., 2018a).

Conducting an initial trauma survey in the field is inherently complex, particularly in cases involving head and neck injuries (Alali et al., 2018). One well-established TBI biomarker is the pupillary light reflex (PLR). The reflexive constriction of the pupil in response to a flash of light is a direct representation of the functional state of the central nervous system (Dewan et al., 2018b; GBD, 2019; Gurney et al., 2020). The PLR can indicate increased intracranial pressure, a more severe consequence of TBI (Haas et al., 2010), and it has been shown to be abnormal even in concussion (Hall and Chilcott, 2018) and mild TBI (Helmick et al., 2015). The PLR is among the most important early indicators of TBI (Hernández-Sierra et al., 2021), and the simplest and most common method of PLR assessment is the traditional penlight exam (also known as manual pupillometry), in which a handheld light source is used to elicit constriction of the pupil. The examiner then uses the naked eye to determine the extent and nature of the PLR. While simple and affordable, this method lacks inter-observer reliability (GBD, 2019). Digital pupillometry currently represents the gold standard for assessing the PLR (Larson and Behrends, 2015); however, such machines are expensive and require specialized training to use.

**FIGURE 1:**
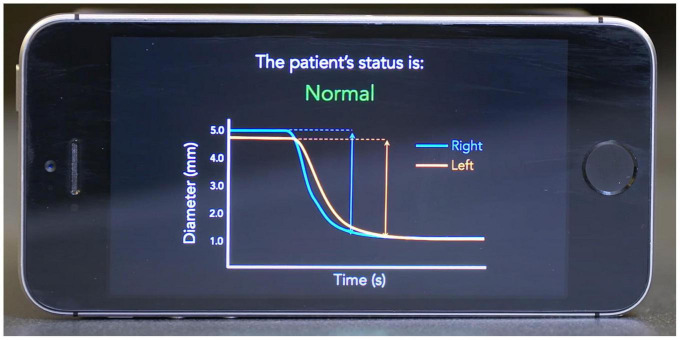
Example mockup screen from the PupilScreen application. A pupillary light reflex (PLR) curve is shown with constriction demonstrating a normal healthy result based on the curve morphology.

To address the shortcomings of current clinical pupillometry techniques, we developed a mobile application called PupilScreen (Mariakakis et al., 2017; Figure 1). PupilScreen is a machine learning-driven application that relies upon computer vision neural network algorithms and is designed to perform pupillometry on the smartphone platform to provide a method of assessing the PLR with more accuracy and reliability than manual pupillometry, while being more accessible than digital pupillometry. While a previously published study demonstrated the accuracy of the application in assessing the PLR (Mariakakis et al., 2017), the best method for presenting these results to the examiner for interpretation remains unclear. The aim of this study is to determine whether practitioners can assess PLR normalcy simply by viewing the PLR curve generated by PupilScreen, and to compare the interrater reliability of this method of assessment to the more traditional penlight method. Alternative methods for smartphone detection of the PLR do exist (Meeker et al., 2005; McAnany et al., 2018; Master et al., 2020), however PupilScreen is currently unique in its binocular approach to measuring the PLR.

## Materials and Methods

**FIGURE 2:**
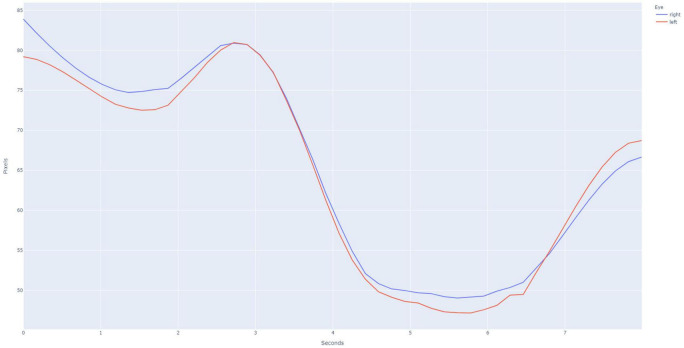
Example pupillary light reflex (PLR) curve for a normal subject, as is evidenced by the large amount of constriction after the light stimulus is applied at the 3 s time point. Binocular recording of the PLR is obtained using the PupilScreen application such that the simultaneous constriction of both the right eye (blue curve) and the left eye (red curve). The current generation of PupilScreen produces PLR curves in terms of pixels and seconds.

**FIGURE 3:**
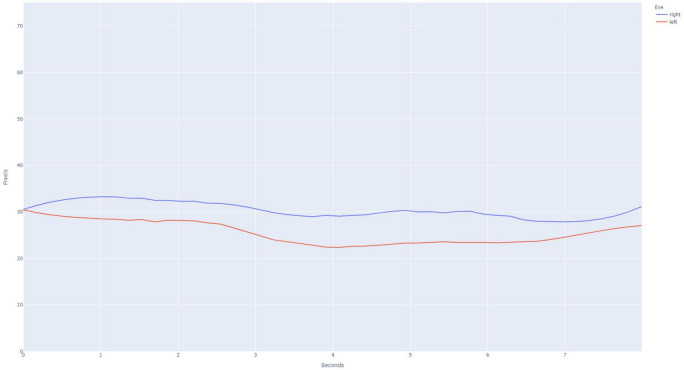
Example pupillary light reflex (PLR) curve for an abnormal subject, as is evidenced by the lack of constriction after light stimulus is applied at the 3 s time point. The PLR curves for the right eye (blue) and left eye (red) are presented.

**FIGURE 4:**
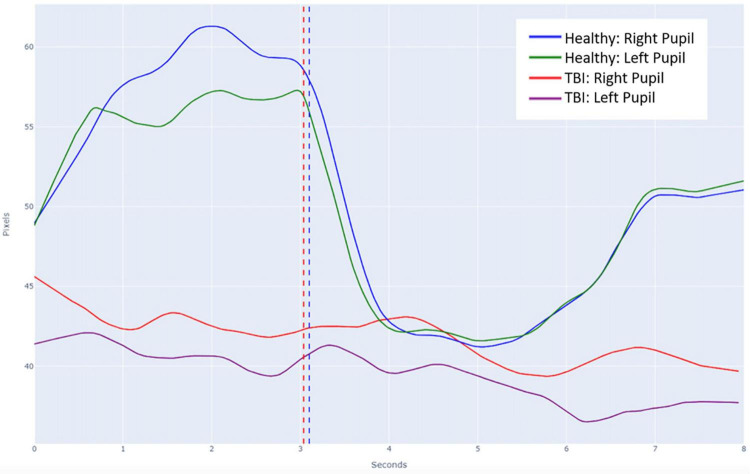
Average pupillary light reflex (PLR) curves overlayed on a single plot for both normal and abnormal subjects. The vertical dashed line in blue represents the timing of the light stimulus for the normal healthy recordings and the vertical dashed line in red represents the slightly differential timing of the light stimulus for the abnormal TBI recordings.

This study was approved by the University of Washington’s Institutional Review Board. A database of individually validated normal and abnormal PLR curves was generated through a process employed in the initial development of the PupilScreen app and as previously detailed (Mariakakis et al., 2017). In short, 42 healthy individuals (Table 1) with a variety of pupillary sizes, iris colors and skin tones were recruited for study. Two predicted PLR curves were generated from each subject representing 84 total normal curves, with curve normality determined by the reactivity of the pupil evident via the curve morphology (Figure 2). Additionally, six patients with severe TBI (as determined both clinically and with the gold-standard digital pupillometer) and subsequently deranged PLR—as evidenced in these patients with severe TBI by a lack of pupillary constriction evident via curve morphology—were recruited during their stay in a neurological intensive care unit alongside the typical use of pupillometry in the care of these patients at our institution, and each generated six samples totaling 36 abnormal predicted PLR curves (Figure 3). A random selection of 24 PLR curves from both the normal and abnormal PLR groups were combined to form one master set of 48 randomly selected and deidentified PLR curve samples. Average normal and abnormal PLR curves are provided as a reference for the reader (Figure 4).

**TABLE 1:** Characteristics of healthy volunteers for building sample PLR curves.

Volunteer characteristics	N (%)
Male	16 (38.1%)
Female	26 (61.9%)
**Iris color**	
Blue	17 (40.5%)
Brown	20 (47.6%)
Mixed	5 (11.9%)

Clinicians were chosen to assess the different groups’ PLR curves from a pool of providers working in the neuro-intensive care unit (NICU) who regularly assess patient’s pupillary responsiveness, both with and without digital pupillometers. All selected nurses were members of the NICU team who regularly care for and monitor neurosurgical and neurology patients. All selected physicians were residents specializing in either neurological surgery, neurology, or anesthesia. These participants were asked to interpret the PLR curves without any additional instruction or patient information. They classified each curve as “normal” or “abnormal.” Sorted curves were then scored as correct or incorrect by a member of the research team.

Statistical analysis was performed to assess individual accuracy, sensitivity, and specificity. A Cohen’s kappa coefficient (*k*) was calculated for each individual practitioner to determine the degree of agreement between the individual practitioner’s assessments and the correct assessments. The entire cohort was then analyzed for diagnostic consistency. Randolph’s kappa coefficient (*K*), an extension of Cohen’s kappa in groups of greater than two raters, was calculated within all nurses, all physicians, and the entire clinician cohort.

## Results

Six physicians and five nurses served as raters for the study (Table 2). Nurses accurately classified the PLR curves as “normal” or “abnormal” at a rate of 91%, physicians at a rate of 94% and the entire group at a rate of 93%. Sensitivity was calculated as a measure of the test’s ability to rule out an abnormality through correct identification of a normal curve. Sensitivity of the test as interpreted by nurses, physicians, and the entire group was 88, 98, and 94%, respectively. Positive predictive value, or the ability to determine that an abnormal PLR was abnormal, was 93, 91, and 92% for nurses, physicians, and the entire group, respectively. Conversely, specificity was calculated as a measure of the test’s ability to rule in an abnormality by virtue of correctly identifying abnormal curves. Nurses, physicians, and the entire group demonstrated specificity of 93, 90, and 92%, respectively. The negative predictive value was 89% for nurses, 98% for physicians, and 93% for the entire group.

**TABLE 2:** PupilScreen measures of diagnostic accuracy.

	Accuracy	Sensitivity	Specificity	Positive predictive value	Negative predictive value	Cohen’s Kappa
**Nurses**	**91%**	**88%**	**93%**	**93%**	**89%**	**0.82**
1	92%	100%	83%	86%	100%	0.83
2	83%	71%	96%	94%	77%	0.67
3	96%	96%	96%	96%	96%	0.92
4	88%	75%	100%	100%	80%	0.75
5	96%	100%	92%	92%	100%	0.92
**Doctors**	**94%**	**98%**	**90%**	**91%**	**98%**	**0.88**
1	75%	96%	54%	68%	93%	0.50
2	96%	92%	100%	100%	92%	0.92
3	100%	100%	100%	100%	100%	1.00
4	96%	100%	92%	92%	100%	0.92
5	100%	100%	100%	100%	100%	1.00
6	98%	100%	96%	96%	100%	0.96
**Overall**	**93%**	**94%**	**92%**	**92%**	**93%**	**0.85**

Reliability within each provider group was high: *k* > 0.8 across groups (nurses *k* = 0.82, physicians *k* = 0.88, total *k* = 0.85). In terms of consistency (Table 3), *K* demonstrated substantial interrater reliability between all nurses (*K* = 0.72), between all physicians (*K* = 0.78), and between the entire group (*K* = 0.75).

**TABLE 3:** PupilScreen measures of diagnostic consistency (inter-rater reliability).

	N	Agreement	Randolph’s Kappa
**Nurses**	**5**	**85%**	**0.72** (0.63, 0.81)
**Doctors**	**6**	**89%**	**0.78** (0.71, 0.86)
**Overall**	**11**	**88%**	**0.75** (0.71, 0.79)

*Bold values represent the Randolph’s kappa (with 95% confidence interval in parentheses below each bold value). And agreement between members of that group (i.e., nurses had overall agreement of 85% in their diagnoses with Randolph’s kappa of 0.72).*

## Discussion

In the present study we seek to understand the potential of a novel method of data presentation to enhance provider accuracy in evaluating the PLR generated by a smartphone-based pupillometry app. The output provided by PupilScreen is a two-dimensional representation of the PLR curve itself, as opposed to a single number calculated by software, such as the Neurological Pupil Index that is reported by digital pupillometers (Okie, 2005). In this proof-of-concept study, we demonstrated that clinicians could interpret these curves based solely on the appearance of the graphed PLR, without any further calculation or interpretation. This is an important first step in crafting digital health tools in a way that maximizes value and minimizes cost and complexity.

This study showed that physicians and critical care nurses were able to interpret the PLR curves generated by the PupilScreen app with a high degree of accuracy, which compares favorably with current standard pupillometry assessments (Olson et al., 2017). These results confirm our previous work (Mariakakis et al., 2017) which had preliminarily indicated that providers can interpret PLR curves solely based on their two-dimensional morphology. In our study, all raters were able to interpret the PLR curves in this format to determine whether the patients’ pupils were reactive or non-reactive with an accuracy of 88% or better. Both the sensitivity and the specificity of the test were high, implying that the test is useful for both identifying an abnormally non-reactive pupil, and for excluding an abnormality by confirming normal reactivity. The kappa coefficients were also high, confirming that PupilScreen maintained high interrater reliability. These results compare favorably to manual pupillometry (Olson et al., 2017).

Currently there are two methods used to assess PLR in the clinical setting: digital infrared pupillometry and manual pupillometry. Manual pupillometry is a low-tech, low-cost, portable approach that remains the standard of practice in most clinical settings both in the US and globally (Olson et al., 2017). Manual pupillometry is highly subjective and has been shown to be imprecise, with a median error of 0.5 mm, more than twice that of a digital infrared pupillometer (Papageorgiou et al., 2008; Olson et al., 2016, 2017; Prabhakaran et al., 2017; Ong et al., 2019). In contrast, PupilScreen can measure the pupil diameter with a median error of 0.36 mm, an improvement over manual pupillometry (Mariakakis et al., 2017) and the curve interpretation that PupilScreen provides allows for further improved accuracy over manual penlight pupillometry. In addition, specific, useful components of the PLR, such as constriction velocity and amplitude (Ong et al., 2019), cannot be quantified with manual pupillometry. Instead, pupillary reactivity is expressed in general terms such as “normal,” “sluggish,” or “fixed.” These qualitative metrics are notoriously unreliable across examiners: In one single-blinded observational study of 2,329 paired assessments, interobserver agreement was moderate-to-poor for pupil size (κ = 0.54) and reactivity (κ = 0.40). Importantly, only 33.3% of the pupils judged by to be non-reactive (an indicator of severe TBI) were found to be non-reactive when further assessed by digital infrared pupillometry (Olson et al., 2017). Another study that compared trained healthcare professionals’ assessment of the PLR vs. a pupillometer showed discordance of 18% (Olson et al., 2016), demonstrating that even with extensive training, the subjective method of measuring the PLR without digital pupillometry may yield inaccurate data.

Digital infrared pupillometry is the current clinical gold standard, consisting of a device with a light-emitting diode (LED) light source and a digital infrared camera. The device utilizes infrared imaging to detect the boundaries of the pupil, then stimulates the eye with a flash from the LED source, eliciting and tracking the pupillary diameter constriction and calculating a Neurological Pupil Index score. Digital infrared pupillometry can successfully track pupil diameter with a median error of 0.23 mm (Prabhakaran et al., 2017). For comparison, the median resting pupil diameter of a healthy patient is about 3.4 mm and constricts by about 0.88 mm with light exposure of 180–200 cd/m (Ravindra et al., 2015). Digital infrared pupillometry is reliable in regular clinical use; however, the method suffers from disadvantages that limit widespread adoption outside of high-resource settings. Digital infrared pupillometer devices are expensive and require disposable parts for each patient, leading to ongoing operational cost. They are not portable and require a power source. Additionally, providers must be trained in both the acquisition and interpretation of results.

Given the growing global burden of TBI, a tool that bridges the accessibility of the penlight exam with the precision and accuracy of the digital pupillometer fills a critical need. While quick and accurate triage is important in routine pre-hospital care of all trauma patients with a possible head injury, providing this sort of capability is even more critical in low-and-middle income countries in which about 90% of the world’s trauma occurs (Scott et al., 2005), and which simultaneously suffer from a chronic insufficiency of both human and technical resources, particularly for advanced subspecialties such as neurosurgery (Stiver and Manley, 2008; Shoyombo et al., 2018). In modern war zones, blast injuries routinely cause severe TBI (Taylor et al., 2003). It is estimated that TBI has affected an estimated 22% of those injured in recent conflicts in Iraq and Afghanistan (Thurman et al., 1998). Management of these injuries is extremely difficult, in part due to the innate scarcity of technical medical resources in these environments and the extreme costs involved in deploying any such equipment in terms of both added risk and dollars (Warf, 2015; Velez et al., 2020). In the United States, sports-related or mild TBI also represents a growing concern. Approximately 300,000 sports-related TBIs occur annually, although this is thought to be an underestimate as it is estimated that half of all such diagnoses are currently missed (Xiang et al., 2014), for example as a result of reactive but abnormal pupils. These patients are most often evaluated via qualitative testing on the sidelines rather than in a well-equipped clinical setting. The PupilScreen technology demonstrated in this study has the future potential to address some of the shortcomings of existing methods of TBI assessment in these diverse environments and patient populations, such as reactive but abnormal pupils that may be nuanced and difficult to differentiate using subjective manual penlight pupillometry.

Strengths of this study include a robust statistical analysis of practitioners that specifically represent the primary end users for this technology. This study was limited by the relatively small number of clinician raters. While only resident physicians and nurses were tested, we would expect this to be useful to a broader spectrum of providers, including first responders, nurse practitioners, and medical assistants. Additionally, those tested regularly assess pupils with either a penlight exam or pupillometry; we did not assess the ability of raters who do not perform pupil assessment regularly. Lastly, it will be important to validate the utility of the PupilScreen PLR curves in patients with mild and moderate TBI in addition to healthy volunteers and patients with severe TBI (as in the current study), as differences in the PLR curve will likely be more subtle and potentially more difficult to appreciate in those cases. A future goal of study using the PupilScreen technology is to demonstrate the ability to differentiate such subtle differences in the acute concussion or mild TBI populations. Furthermore, future research will focus on broadening participation to multiple types of practitioners and to include results from patients spanning the full spectrum of TBI.

## Conclusion

In conclusion, the PupilScreen app generated PLR curves from normal subjects and TBI patients that were accurately interpreted as normal or abnormal by physician and nurse raters without prior training. The level of accuracy and interrater reliability was higher than those of historical studies using manual pupillometry, which may improve future clinical management of TBI patients. This demonstrates the potential for machine learning-driven mobile digital health technologies to improve TBI diagnosis in a variety of clinical environments.

## Data Availability Statement

The raw data supporting the conclusions of this article will be made available by the authors, without undue reservation.

## Ethics Statement

The studies involving human participants were reviewed and approved by the University of Washington Institutional Review Board. The patients/participants provided their written informed consent to participate in this study.

## Author Contributions

LM was responsible for software development, data collection, and data analysis. JE and IA were responsible for data collection and manuscript writing. AM was responsible for data processing and analyses and manuscript revisions. CK was responsible for data analysis, manuscript writing, and revisions. RC and ML were responsible for data interpretation, manuscript writing, and revisions. All authors contributed to the article and approved the submitted version.

## Conflict of Interest

LM was a cofounder and employee of EigenHealth Inc., the creators of the PupilScreen application. ML was a consultant for Medtronic and Metis Innovative, and has equity interest in Synchron, Cerebrotech, and Proprio. The remaining authors declare that the research was conducted in the absence of any commercial or financial relationships that could be construed as a potential conflict of interest.

## Publisher’s Note

All claims expressed in this article are solely those of the authors and do not necessarily represent those of their affiliated organizations, or those of the publisher, the editors and the reviewers. Any product that may be evaluated in this article, or claim that may be made by its manufacturer, is not guaranteed or endorsed by the publisher.
